# Multiferroic Studies of RMnO_3_ [R = Y, Er, Yb] Hexamanganites Thin Films

**DOI:** 10.1021/acsomega.5c06836

**Published:** 2026-07-02

**Authors:** Inchara D. R, Mamatha D. Daivajna

**Affiliations:** Department of Physics, Manipal Institute of Technology, 125853 Manipal Academy of Higher Education, Manipal 576104, Karnataka, India

## Abstract

Stable, nontoxic, and cost-effective hexagonal RMnO_3_ (R = Y, Er, Yb) thin films were synthesized via spray pyrolysis and optimized for structural quality. The hexagonal structure and dense surface morphology were confirmed by X-ray diffraction (XRD) and scanning electron microscopy (SEM), consistent with our previous study. Detailed magnetic and ferroelectric characterizations were carried out. All films exhibited clear paramagnetic-to-antiferromagnetic (AFM) transitions. The Neel temperature of all the samples was determined. Room temperature Polarization–Electric field (P–E) hysteresis loops confirmed leaky ferroelectric behavior, influenced by internal bias fields and defect structures. The results demonstrate the coexistence of magnetic and ferroelectric orderings in stable oxide multiferroic material RMnO_3_ thin films, underscoring their potential as multiferroic materials for future applications in magnetoelectric sensors, nonvolatile memories, and spintronic devices.

## Graphical abstract

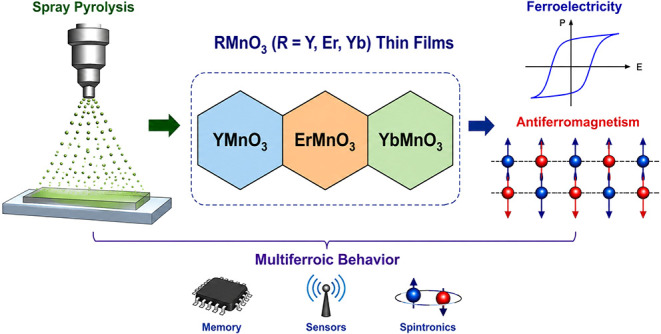

## 1 Introduction

The search for materials that show the capability of exhibiting multiple ferroic orders (ferroelectricity/ferromagnetism/ferroelasticity) has led to a great deal of investigation of multiferroic oxides, especially given the potential of these materials in multifunctional devices that process logic, memory, and sensing functions in one material platform.^1^ Multiferroic materials are attractive, especially in applications where electric polarization is driven by a magnetic field, or magnetization is driven by an electric field, as they could potentially lead to energy-efficient spintronic and magnetoelectric devices.^2,3^ However, it is difficult to realize strong ferroelectric and magnetic orderings at the same time in the same phase due to the commonly reciprocal electronic requirements.

Recent years have seen significant progress in the development of molecular multiferroics, which leverage the flexibility and tunability of molecular and hybrid structures to achieve multiferroic behavior at room temperature. These materials, including organic–inorganic hybrids, metal–organic frameworks, and layered two-dimensional systems, exhibit coupled electric and magnetic orders through inherent molecular dipoles and spin interactions. Recent studies have demonstrated dielectric, ferroelectric, and magnetoelectric properties with strong tunability via chemical design, strain engineering, and external fields. Such advances extend the multiferroic paradigm beyond oxide-based systems, offering promising platforms for low-power, multifunctional devices, including flexible electronics, sensors, and spintronic applications. Recent studies have advanced the understanding of magnetoelectric coupling mechanisms in multiferroics, highlighting emergent phenomena driven by spin–phonon interactions and structural engineering.^4^ Additionally, novel insights into enhanced and robust magnetoelectric effects in hybrid multiferroic heterostructures have been reported, offering new opportunities for nanoscale device applications.^5^

Among the limited number of materials that fulfill these criteria, hexagonal rare-earth manganites (RMnO_3_, where R = Y, Er, Yb, etc.) have emerged as prototypical type I multiferroics, in which ferroelectricity and magnetic orderings have distinct microscopic origins.^6^ These compounds crystallize in the noncentrosymmetric *P*6_3_ *cm* space group, where the lattice undergoes a high temperature transition to a ferroelectric phase driven by a geometric structural distortion rather than conventional cation off-centering.^8^ This distortion involves a trimerization of MnO_5_ bipyramids and the buckling of R^3+^ layers, resulting in spontaneous electric polarization along the hexagonal *c*-axis. The origin of ferroelectricity in these materials is thus classified as improper or geometric, fundamentally distinct from classical perovskite ferroelectrics.^8,9^

The ferroelectric transition temperature (*T*_c_) in hexagonal RMnO_3_ is typically very high (≈1000 K), enabling room temperature functionality and making them suitable for integration in ferroelectric-based devices.^10^ The ferroelectric properties arise from the off-centering of the R ions, leading to spontaneous electric polarization, while the magnetic properties are due to the ordering of Mn^3+^ spins in the antiferromagnetic (AFM) arrangement. In contrast, antiferromagnetic ordering of the Mn^3+^ (3d^4^, *S* = 2) sublattice occurs at much lower temperatures (*T*_N_ ≈ 30–80 K), arising from superexchange interactions through the Mn–O–Mn network in the ab-plane.^11^ The degree of Mn–O–Mn bond angle distortion, modulated by the size of the R^3+^ cation, critically affects the magnetic ordering temperature and strength of the antiferromagnetic exchange.^12^ In some cases, the R-site rare-earth ions themselves carry localized magnetic moments, contributing to additional spin interactions with the Mn sublattice and further complicating the magnetic ground state.^13^ The decoupled origins of ferroelectric and magnetic orderings in these systems result in weak direct magnetoelectric coupling in the bulk.

Recent work has shown that ferroelectric domain walls and vortex core structures can exhibit strong coupling which is topologically protected and which permits emergent functionalities that are not found in the homogeneous bulk phase.^14^ These vortex domain structures were first imaged in YMnO_3_ and originate from the anisotropic coupling between the trimerized lattice distortion and polarization vector fields, forming cloverleaf domain patterns.^15^ The presence of these cloverleaf shapes makes hexagonal manganites particularly interesting systems for the study of topological defects, domain-wall engineering, and emergent phenomena in low-dimensional ferroics. Additionally, hexagonal manganites exhibit interesting phenomena, such as topological defects, where the polarization vortices and domain walls can have unique physical properties. The coexistence of electric and magnetic orders in the same material opens up possibilities for novel device applications where electric fields can control magnetic properties and vice versa.^16^ These characteristics make hexagonal manganites a rich subject for fundamental research as well as for exploring new technological applications in the field of multiferroics.^17^ This material has attracted significant attention due to its potential applications in nonvolatile memory devices, spintronics, and sensors.^12^ The interplay between its ferroelectric and magnetic orders at relatively high temperatures makes it a promising candidate for multifunctional devices.

Thin films offer the additional advantage of tunability via strain, substrate interaction, and film thickness.^18^ In particular, spray pyrolysis has emerged as a simple, scalable, and cost-effective method for synthesizing oxide thin films with good control over stoichiometry and phase purity.^19^ However, detailed investigations into the structure–property relationships of RMnO_3_ thin films synthesized via spray pyrolysis remain limited, especially for the lesser-explored compositions such as ErMnO_3_ and YbMnO_3_.

In this study, we present the synthesis and characterization of RMnO_3_ (R = Y, Er, Yb) thin films, fabricated by spray pyrolysis and optimized accordingly and under appropriate processing conditions. The main objective of this research is to systematically investigate the multiferroic properties of these compositions with temperature-dependent magnetic susceptibility, magnetization hysteresis, and ferroelectric polarization–electric field (P–E) loop measurements at room temperature. By comparing the structural with magnetic/ferroelectric properties, this study provides insight into furthering the understanding of hexagonal manganite thin films and demonstrating their potential for practical magnetoelectric devices.

## 2 Experimental Details

### 2.1 Preparation of Precursor Solutions and Films

The hexamanganites YMnO_3_, ErMnO_3_, and YbMnO_3_ were prepared using the spray pyrolysis technique on the quartz substrate. The detailed synthesis procedure and optimization conditions are reported in our earlier work.^20^

In that study, detailed structural and morphological characterization confirmed the formation of single-phase hexagonal (*P*6_3_cm) RMnO_3_ films (not shown here). X-ray diffraction (XRD) analysis verified their crystallinity and phase purity, while scanning electron microscopy (SEM) revealed dense, smooth, and crack-free surfaces with homogeneous grain morphology in the 80–120 nm range. Considering these confirmed features, only the optimized films were selected for further magnetic and ferroelectric characterization in the present study.

### 2.2 Characterization

Magnetic characterization was performed using a superconducting quantum interference device (SQUID) magnetometer (Quantum Design MPMS). Temperature-dependent magnetic susceptibility was measured from 5 to 300 K under an applied magnetic field of 100 Oe. Magnetic hysteresis (M–H) measurements were carried out at low temperatures under magnetic fields ranging from 0 to +5 T to study the magnetic response of the films. Ferroelectric measurements were conducted at room temperature utilizing a P–E loop tracer (Radiant Technology, USA). Polarization–electric field (P–E) hysteresis loops were obtained at applied DC voltages of 50 and 100 V. Key ferroelectric parameters such as remanent polarization (*P*_r_), saturation polarization (*P*_s_), coercive field (*E*_c_), internal bias field (*E*_int_), and horizontal shift were extracted from the loops.

## 3 Results and Discussion

### 3.1 Magnetisation Studies

Temperature-dependent magnetic susceptibility, its first derivative, and magnetic field-dependent magnetization (M–H) measurements were carried out to investigate the magnetic behavior of RMnO_3_ (R = Y, Er, Yb) thin films, and these plots of all the samples are presented in Figures 1–3(a–c), respectively. The zero-field-cooled (ZFC) and field-cooled (FC) magnetization curves were recorded under a 100 Oe field in the temperature range of 5–300 K. The bifurcation observed in ZFC and FC curves indicates the onset of magnetic ordering and confirms the transition from a paramagnetic to an antiferromagnetic state. The temperature at which this divergence occurs is associated with the Néel temperature (*T*_N_), a hallmark of antiferromagnetic materials.^12^

**Figure 1:**
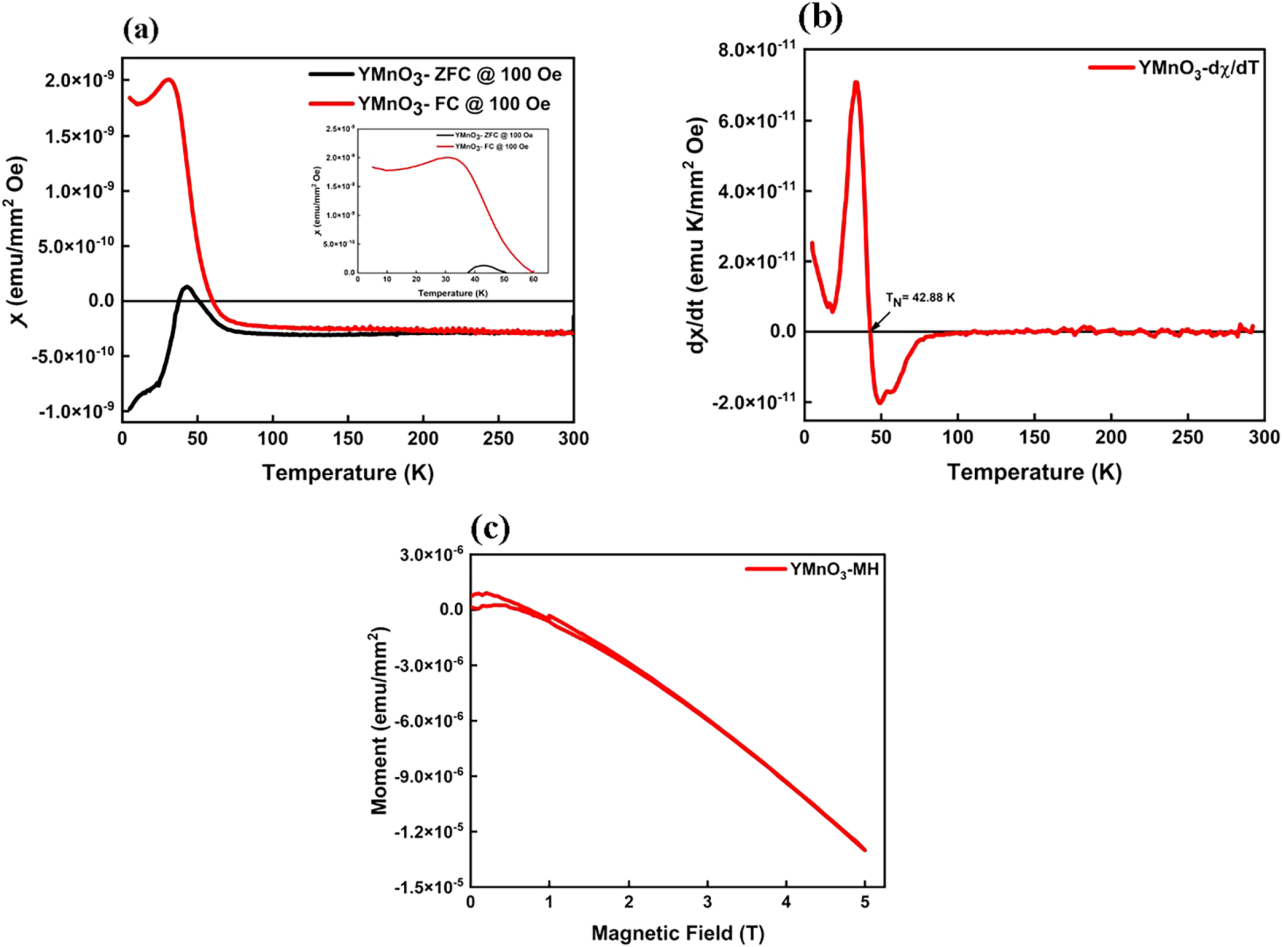
(a) Temperature-dependent susceptibility of YMnO_3_. (b) First-order derivative of the susceptibility of YMnO_3_. (c) Magnetic-field-dependent magnetization of YMnO_3_.

**2:**
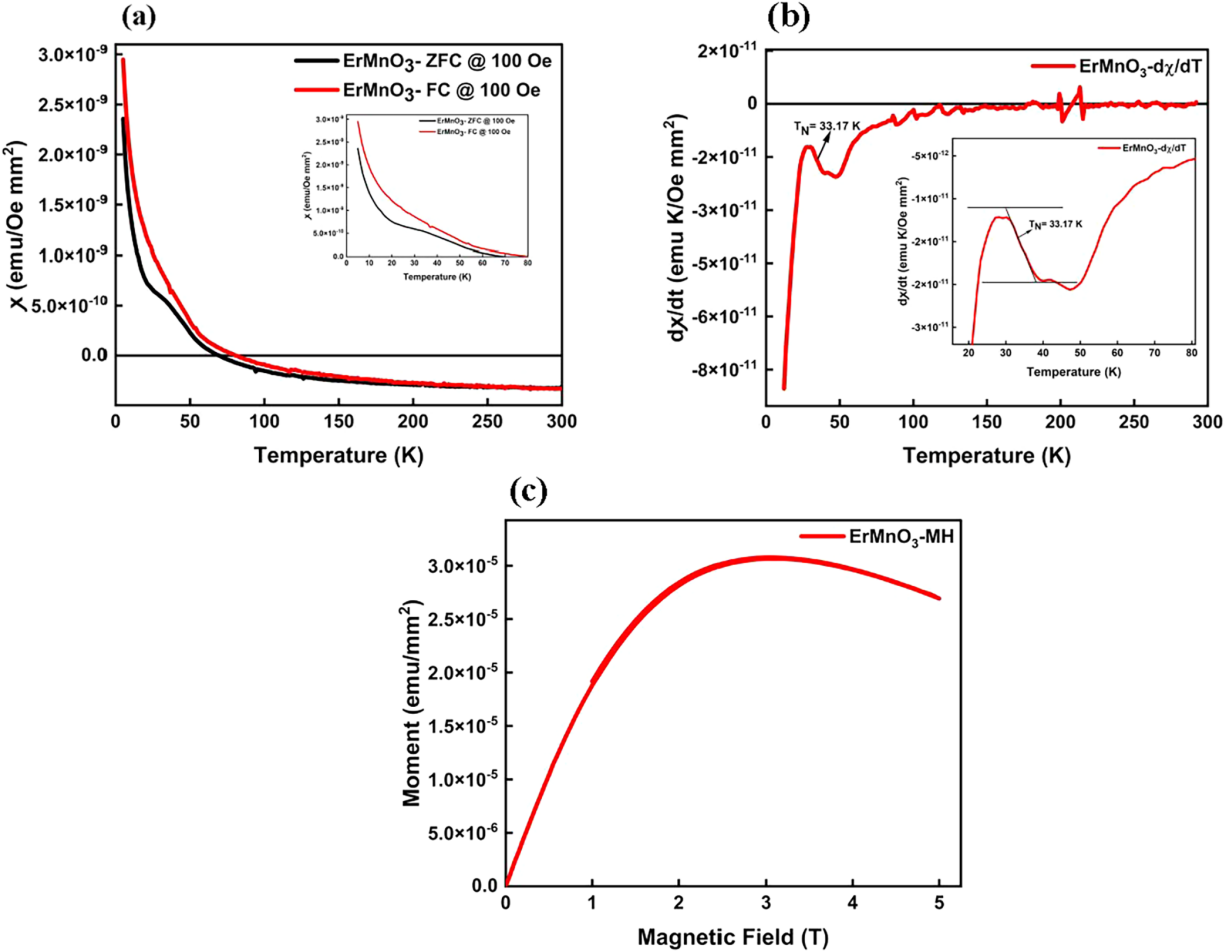
(a) Temperature-dependent susceptibility of ErMnO_3_. (b) First-order derivative of the susceptibility of ErMnO_3_. (c) Magnetic-field-dependent magnetization of ErMnO_3_.

**Figure 3:**
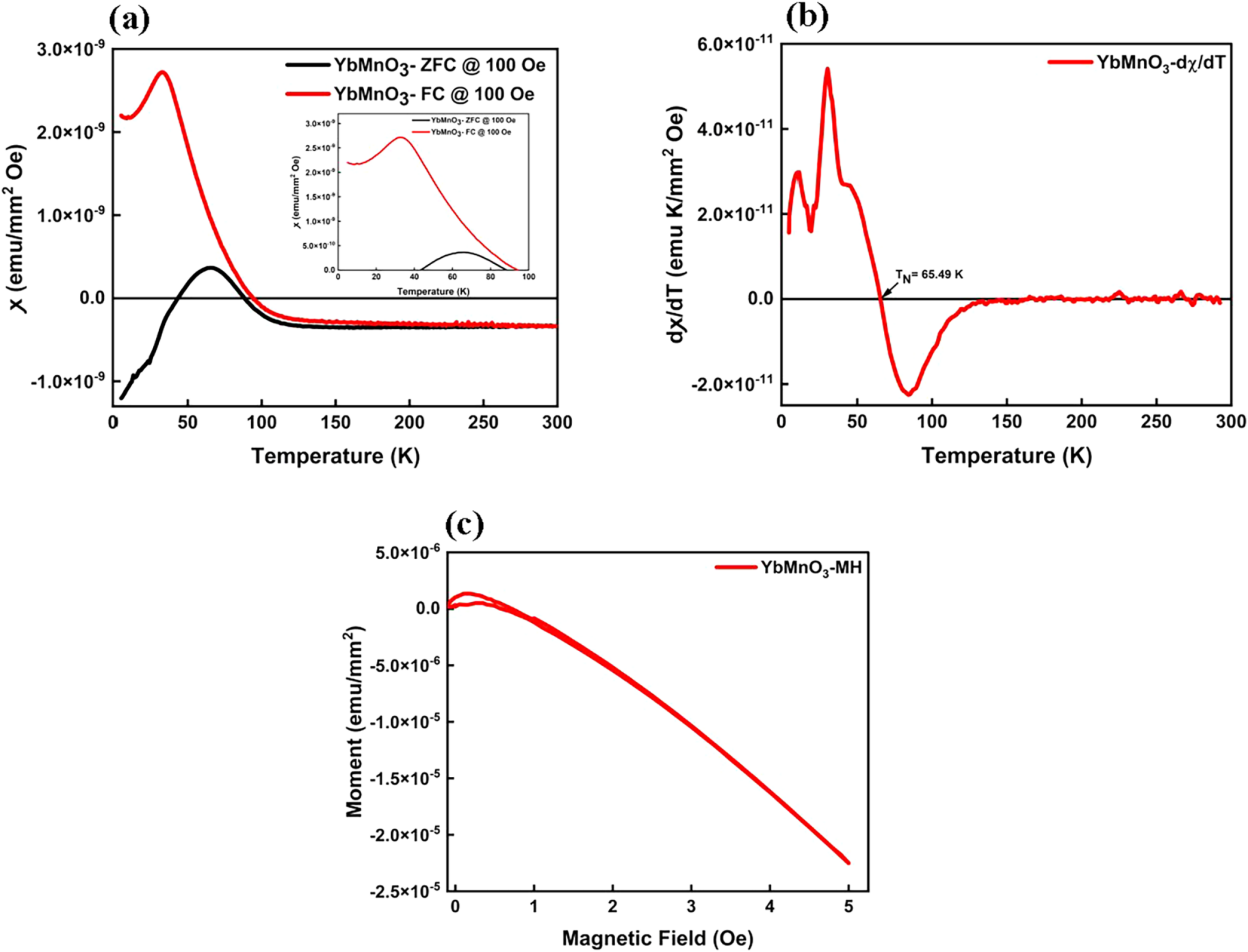
(a) Temperature-dependent susceptibility of YbMnO_3_. (b) First-order derivative of the susceptibility of YbMnO_3_. (c) Magnetic-field-dependent magnetization of YbMnO_3_.

A sharp rise in magnetic susceptibility with decreasing temperature, followed by a decrease below some critical temperature (Néel temperature, *T*_N_), was found for YMnO_3_ and YbMnO_3_. This is the characteristic of the paramagnetic to antiferromagnetic (AFM) transition, in which increased spin correlations between Mn^3+^ ions (3d^4^, *S* = 2) in the vicinity of *T*_N_ result in an increase in susceptibility that later decreases as long-range AFM order establishes.^11,12^ In YMnO_3_, the effect is particularly clear because magnetic R-site ions are absent—Y^3+^ being nonmagnetic; thus, the Mn^3+^ sublattice determines the overall magnetism. In YbMnO_3_, while Yb^3+^ has a magnetic moment, its contribution is only important at extremely low temperatures, and Mn^3+^ ordering is still predominant at *T*_N_. The narrowness of the transition in these compounds demonstrates a comparatively clear-cut beginning of antiferromagnetic spin order.^22^

Conversely, ErMnO_3_ does not show a brusque drop in susceptibility below *T*_N_. This is due to the high magnetic moment of Er^3+^ ions (∼9.6 μB) and the strong 3d–4f coupling of the latter with the Mn^3+^ sublattice. Er^3+^ moments are independently ordering their moments at much lower temperatures (∼2.5 K), but their high magnetic contribution smooths the susceptibility in a wide temperature range, which hides or broadens the Mn-related transition.^22,23^ In addition to that, crystal field effects and magnetic anisotropy due to the Er^3+^ ions improve the smoother magnetic response. These types of interactions make the spin dynamics complicated and avoid a clear signature in susceptibility, contrasting with the sharp features present in YMnO_3_ and YbMnO_3_. This suggests a smoother evolution of magnetic order without abrupt changes in susceptibility. Negative susceptibility values at low temperatures are attributed to the diamagnetic contribution from the quartz substrate.^20^ That is because the thickness of the substrate is more than the thickness of the film, due to which the diamagnetic nature dominates. In the first-order derivative of susceptibility plots of YMnO_3_, ErMnO_3_, and YbMnO_3_ shown in Figures 1–3 (a), respectively, the temperature where the slope is zero (dχ/d*T* = 0) marks *T*_N_. For YMnO_3_, the Néel temperatures were determined to be 42.88 K; for ErMnO_3_, 33.17 K; and for YbMnO_3_, 65.69 K, respectively.

The Mn^3+^ and Mn^4+^ ions’ superexchange interaction is thought to be the cause of the antiferromagnetic property. The virtual transfer of electrons between two nearest neighboring Mn ions having the same valency happens via an oxygen ion. The planes can end up in a frustrating arrangement as a result.^11,21^ Moreover, the magnetic moments of R^3+^ ions such as Er^3+^ and Yb^3+^ can interact with the Mn^3+^ sublattice via 3d–4f coupling, so affecting the total magnetic behavior.^22^ The strength of this interaction can also vary with the ionic radius, as the spatial distance and alignment between R^3+^ and Mn^3+^ sublattices are affected which leads to a change in Neel temperature.

Figures 1–3 (c) show the M-H plots of YMnO_3_, ErMnO_3_, and YbMnO_3,_ respectively, from 0 to 5 T measured at 5 K. Due to the antiferromagnetic nature of the RMnO_3_ films, the magnetization exhibits a symmetric, reversible, and linear response with minimal hysteresis. Therefore, the magnetization was measured from 0 to 5 T, which adequately captures the field-dependent behavior. Negative field measurements would not provide additional magnetic information and were excluded to reduce noise and substrate interference. Indicative of antiferromagnetic ordering with a strong diamagnetic background, YMnO_3_ and YbMnO_3_ displayed almost linear magnetization curves with negligible hysteresis. In such systems, the Mn^3+^ spins are coupled through superexchange interactions along Mn–O–Mn bridges, resulting in antiparallel spin alignment with zero net magnetization, and therefore no hysteresis loop.^11,12^ The absence of a magnetic contribution from R-site ions in YMnO_3_ (nonmagnetic Y^3+^) and the restricted impact of Yb^3+^ moments at 5 K guarantee that the magnetic response continues to be controlled by AFM Mn^3+^ order.

On the other hand, ErMnO_3_ shows a clear hysteresis loop at the same temperature, reflecting a weak ferromagnetic component superimposed on the AFM base. This is presumably caused by spin canting of the Mn^3+^ sublattice or magnetic interaction between Er^3+^ and Mn^3+^ ions, disrupting perfect antiparallel alignment. A likely cause of this canting is the Dzyaloshinskii–Moriya (DM) interaction, which occurs in systems without inversion symmetry and can produce a small net moment even in weakly AFM structures.^12,23^ Also, Er^3+^ ions have a high magnetic moment, and strong spin–orbit coupling introduces major magnetic anisotropy that stabilizes the canted spin arrangement, hence the loop opening observed.^22,23^

In addition to the distinct variations observed in *T*_N_ and M–H behavior across the RMnO_3_ series, subtle differences in magnetic relaxation behavior were also noted. The marginal decrease and broadening of *T*_N_ in RMnO_3_ thin films compared to bulk arise due to substrate-induced epitaxial strain, which distorts Mn–O–Mn bond geometry and modifies superexchange interactions. The magnetic relaxation measurements (not shown here) indicated slower magnetic response times in ErMnO_3_ compared to YMnO_3_ and YbMnO_3_, suggesting stronger pinning effects or localized magnetic frustration, possibly due to increased anisotropy introduced by the Er^3+^ ions. These observations point to a complex interplay of 3d–4f interactions, lattice strain, and crystallographic orientation in determining the low-temperature magnetic dynamics of the films. Such behavior could be further explored using AC susceptibility and time-dependent magnetization techniques to better understand the relaxation mechanisms and potential glassy dynamics in ErMnO_3_. These effects, though subtle, contribute to the nuanced magnetic response of hexagonal manganites and add another layer of tunability for future device applications.

These results underscore the function of the R-site substitution in modulating the magnetic properties of RMnO_3_ films. The interplay among lattice distortion, magnetic exchange interactions, and rare-earth magnetic moments offers opportunities to engineer multifunctional materials for spintronic and magnetoelectric device applications.^24^ The summary of the ionic radii and the Neel temperature of all the samples is shown in Table 1.

**Table 1:** Summary of Ionic Radii and *T*_N_ of RMnO_3_ (R = Y, Er, and Yb) Samples

Name of the sample	Ionic radii (Å)	*T*_N_ (K)
YMnO_3_	1.019	42.88
ErMnO_3_	0.890	33.17
YbMnO_3_	0.868	65.49

### 3.2 Ferroelectric Studies

Ferroelectric characteristics of RMnO_3_ (R = Y, Er, and Yb) thin films were studied by polarization–electric field (P–E) loop measurements performed at room temperature. P–E loops, obtained under DC voltages of 50 and 100 V applied, are shown in Figure 4 (a–c) for the respective composition. The applied voltages correspond to electric fields close to the coercive fields necessary for domain switching; however, leakage currents arising from oxygen vacancies and defect structures limited complete polarization reversal. All samples exhibited unsaturated and asymmetric hysteresis loops typical of leaky ferroelectric materials. This leakage is commonly attributed to the presence of defects like oxygen vacancies, which facilitate charge carrier mobility and hinder full polarization switching.^23,25^ Therefore, we interpret the results as exhibiting incipient or leaky ferroelectric behavior rather than ideal ferroelectric hysteresis. Nevertheless, the observed remanent polarization and loop shifts indicate intrinsic ferroelectricity, albeit partially obscured by conduction effects. Future work aimed at improving film quality and optimizing measurement conditions is planned to reduce leakage and enhance ferroelectric switching.

**Figure 4:**
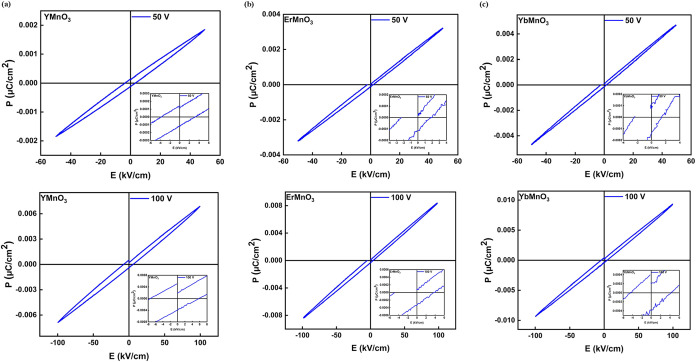
(a) P–E hysteresis loops of YMnO_3_ sample measured at room temperature under different DC voltages.

Insets show enlarged low-field regions highlighting the extraction of remanent polarization (*P*_r_) and coercive field (*E*_c_). *P*_s_ corresponds to the effective polarization attained at the maximum applied field due to unsaturated loop behavior. (b) P–E hysteresis loops of ErMnO_3_ sample measured at room temperature under different DC voltages. Insets show enlarged low-field regions highlighting the extraction of remanent polarization (*P*_r_) and coercive field (*E*_c_). *P*_s_ corresponds to the effective polarization attained at the maximum applied field due to unsaturated loop behavior. (c) P–E hysteresis loops of YbMnO_3_ sample measured at room temperature under different DC voltages. Insets show enlarged low-field regions highlighting the extraction of remanent polarization (*P*_r_) and coercive field (*E*_c_). *P*_s_ corresponds to the effective polarization attained at the maximum applied field due to unsaturated loop behavior. *P*_r_ and *E*_c_ were extracted from the enlarged low-field regions of the P–E loops (insets in Figure 4 (a–c)), corresponding to the polarization at *E* = 0 and the electric field at *P* = 0, respectively. P–E loops were shifted toward the negative voltage axis, which represents the existence of an internal bias field (*E*_int_), which may be due to asymmetric electrode interfaces or defect-dipole alignment in the film matrix.^17,18^ Asymmetry means that there exist pinned domains or built-in electric fields because of localized strain or inhomogeneous charge distribution, possibly at grain boundaries or because of compositional inhomogeneities.^23^ The enhanced loop area that was seen as the voltage increased reflects improved domain switching and polarization orientation despite the lossy character. Importantly, with decreasing ionic radius of the R^3+^ cation down the series (Y > Er > Yb), there was an associated decrease in loop area and remanent polarization. This trend may be linked to the diminished tilting and rotation of MnO_5_ polyhedra, which are responsible for the geometric ferroelectric distortion in hexagonal manganites.^23,26^

Quantitative factors like apparent saturation polarization (*P*_s_), remanent polarization (*P*_r_), coercive field (*E*_c_), internal bias field (*E*_int_), and horizontal shift (δ) were obtained and given in Table 2. Coercive fields were fairly small for all samples, indicating domain switching at reasonable electric fields. However, the small values of *P*_r_ and *P*_s_ further confirm the incomplete domain reversal due to leakage or insufficient crystal alignment.^26^

**Table 2:** List of P–E Loop Parameters of RMnO_3_ (R = Y, Er and Yb) Samples

Sample name	Parameter DC voltage (V)	Apparent *P*_s_ (μC/cm^2^)	*P*_r_ (μC/cm^2^)	Coercive field *E*_c_^+^ (V/cm)	Coercive field *E*_c_^–^ (V/cm)	*E*_int_ (V/cm)	Horizontal shift δ (V/cm)
YMnO_3_	50 V	0.0018	1.3962 × 10^–4^	3.1977	–3.7799	3.4888	–0.5822
YMnO_3_	100 V	0.0069	5.0845 × 10^–4^	5.8317	–7.5353	6.6835	–1.7036
ErMnO_3_	50 V	0.0032	2.5972 × 10^–5^	1.9819	–2.3395	2.1607	–0.3576
ErMnO_3_	100 V	0.0084	7.7581 × 10^–5^	3.9518	–4.9473	4.4495	–0.9955
YbMnO_3_	50 V	0.0047	1.1440 × 10^–4^	1.4355	–2.4237	1.9296	–0.9882
YbMnO_3_	100 V	0.0097	4.0874 × 10^–4^	4.0368	–4.4371	4.2369	–0.4003

These results highlight the central importance of rare-earth ion size in engineering the ferroelectric properties of RMnO_3_ thin films. The correlation among shrinking R^3+^ ionic radius, augmentation of structural distortion, and increased ferroelectric switching fields further solidifies the idea of structural control over ferroic order parameters. This tunability offers an effective avenue for optimizing device-relevant parameters in multiferroic heterostructures, especially for devices with room-temperature functionality such as nonvolatile memories, ferroelectric field-effect transistors, and magnetoelectric sensors.^3,10,16^

This shows noticeable variation across the RMnO_3_ thin films, indicating increased pinning of the domain walls and more vigorous restoring forces in the deformed lattice structure. The greater *E*_c_ values indicate that these films with small R^3+^ ions form stronger internal fields and electrostatic energy barriers to domain reversal, attributed to greater strain, defect density, and electric field gradients locally introduced during the growth process.^15,2,26^ Internal bias fields (*E*_int_) and horizontal loop shifts (δ), shown consistently for all compositions, also reflect asymmetric domain pinning and built-in electric fields, most likely due to charged defects like oxygen vacancies and interface dipoles.^17,18,25^

In hexagonal manganites, ferroelectricity is of geometric or improper type and originates from a structural trimerization of corner-sharing MnO_5_ trigonal bipyramids and a correlated buckling of rare-earth layers, instead of the usual B-site cation displacement characteristic of perovskite ferroelectrics.^7–9^ This trimerization destroys inversion symmetry and pinches in a spontaneous polarization along the *c*-axis of the *P*6_3_ *cm* structure. The extent of this distortion—and thus the ferroelectric polarization—is determined heavily by the ionic radius of R^3+^ ion. The smaller the R-site cation (Y > Er > Yb), the lattice shrinks and increases the tilt and distortion of MnO_5_ units. The structural deformation makes the amplitude of the polar displacement greater and results in higher values of remanent polarization.^8,23,26^

Systematic evolution of ferroelectric behavior is observed throughout the RMnO_3_ thin films as the R-site cation is changed from Y to Er to Yb. This trend is clearly reflected in the remanent polarization (*P*_r_) and coercive field (*E*_c_) values obtained from the P–E hysteresis loops (Table 2). Here, *P*_r_ represents the remanent polarization at zero electric field, expressed in μC/cm^2^, and serves as an indicator of ferroelectric switchability. The calculated polarization values correspond to an electrode area of 0.031 cm^2^ and a film thickness of approximately 280 nm. While the same loops in all cases reveal gross leakage and asymmetry, the relative change in these values emphasizes the effect of R-site substitution on ferroelectric behavior.

The internal bias field was calculated using the relation

1 $$E_{\mathrm{int}}=\frac{E_{c}^{+}-E_{c}^{-}}{2}$$

which provides insight into the direction and magnitude of the built-in electric fields. In these films, the *E*_int_ values varied with composition, further supporting the idea that defect structure and R-site substitution significantly influence ferroelectric behavior.^23,18^

Lossy character and displaced loops are manifestations of competition between ferroelectric domain dynamics and defect-induced pinning effects. Though weak ferroelectricity at ambient conditions is observed in these materials, intrinsic asymmetry and domain pinning can be advantageous for applications like resistive switching and tunable dielectrics, where charge migration is desired in a controlled fashion.^17^

In later work, the films’ quality can be enhanced by annealing, doping techniques, or interface engineering to reduce leakage and prefer higher polarization switching. Furthermore, domain structure studies with piezoresponse force microscopy (PFM) could clarify the actual-space distribution of polarization and its connection to structural properties.^15,18^

Additionally, the microstructure and crystallographic orientation of the RMnO_3_ thin films play a crucial role in determining their ferroelectric performance. It has been reported that grain boundaries and film–substrate interfaces can act as recombination centers or pinning sites for domain walls, which can suppress switchable polarization and contribute to leakage currents.^17,18^ In hexagonal manganites, domain walls themselves can be active entities, with different electrical properties from the bulk material, e.g., increased conductivity or localized reversal of polarization.^15,23^ The vortex like domain structures found in YMnO_3_ and similar compounds also indicate the topologically protected nature of these systems, which can give rise to new memory and logic device functionalities.^23,24^ Thus, texturing the film and reducing defect density by optimized synthesis protocols like controlled spray parameters or postdeposition annealing can further improve ferroelectric response and stability. Furthermore, integrating these thin films into heterostructures or multilayered architectures provides promising avenues to modulate interfacial coupling effects and achieve magnetoelectric devices operating at room temperature.^16,17^ As the hysteresis loops remain unsaturated within the applied field range, *P*_s_ is taken as the polarization attained at the highest applied electric field, rather than a true saturation value.

## 4 Conclusion

Hexagonal RMnO_3_ (R = Y, Er, Yb) thin films were successfully synthesized via spray pyrolysis and optimized based on the parameters of molar concentration of the precursor solution and annealing temperature. Magnetic measurements revealed a paramagnetic-to-antiferromagnetic transition, with Néel temperatures modulated by R-site ionic radii, reflecting the influence of lattice distortion on magnetic ordering. Room-temperature ferroelectric measurements revealed leaky, asymmetrical P–E loops, indicating ferroelectric behavior influenced by internal bias fields and defect structures.

The compatibility of electric and magnetic orderings in these films promises their feasibility for multiferroic device applications. Their tunability with rare-earth substitution promises an auspicious route for embedding RMnO_3_ thin films into spintronic and magnetoelectric devices. Progress toward improved film quality and domain manipulation can provide enhanced functional operation at room-temperature device applications in the future.
